# South Asia’s COVID-19 History and Surveillance: Updated Epidemiological Assessment

**DOI:** 10.2196/53331

**Published:** 2024-08-26

**Authors:** Lori A Post, Alan G Soetikno, Scott A Wu, Claudia Hawkins, Maryann Mason, Egon A Ozer, Robert L Murphy, Sarah B Welch, Yingxuan Liu, Robert J Havey, Charles B Moss, Chad J Achenbach, Alexander L Lundberg

**Affiliations:** 1 Buehler Center for Health Policy and Economics Robert J Havey, MD Institute for Global Health Northwestern University Chicago, IL United States; 2 Department of Emergency Medicine Feinberg School of Medicine Northwestern University Chicago, IL United States; 3 Feinberg School of Medicine Northwestern University Chicago, IL United States; 4 Department of Medicine, Division of Infectious Diseases Feinberg School of Medicine Northwestern University Chicago, IL United States; 5 Center for Global Communicable and Emerging Infectious Diseases Robert J Havey, MD Institute for Global Health Northwestern University Chicago, IL United States; 6 Center for Pathogen Genomics and Microbial Evolution Robert J Havey, MD Institute for Global Health Northwestern University Chicago, IL United States; 7 Robert J Havey, MD Institute for Global Health Northwestern University Chicago, IL United States; 8 Department of Medicine, General Internal Medicine and Geriatrics Feinberg School of Medicine Northwestern University Chicago, IL United States; 9 Institute of Food and Agricultural Sciences University of Florida Gainesville, FL United States

**Keywords:** SARS-CoV-2, COVID-19, South Asia, pandemic history, Bangladesh, Bhutan, India, Maldives, Nepal, Pakistan, Sri Lanka, surveillance, speed, acceleration, jerk, dynamic panel data, generalized method of moments, GMM, Arellano-Bond, 7-day lag

## Abstract

**Background:**

This study updates our findings from the COVID-19 pandemic surveillance we first conducted in South Asia in 2020 with 2 additional years of data for the region. We assess whether COVID-19 had transitioned from pandemic to endemic at the point the World Health Organization (WHO) ended the public health emergency status for COVID-19 on May 5, 2023.

**Objective:**

First, we aim to measure whether there was an expansion or contraction in the pandemic in South Asia around the WHO declaration. Second, we use dynamic and genomic surveillance methods to describe the history of the pandemic in the region and situate the window of the WHO declaration within the broader history. Third, we aim to provide historical context for the course of the pandemic in South Asia.

**Methods:**

In addition to updating the traditional surveillance data and dynamic panel estimates from our original study, this study used data on sequenced SARS-CoV-2 variants from the Global Initiative on Sharing All Influenza Data (GISAID) to identify the appearance and duration of variants of concern. We used Nextclade nomenclature to collect clade designations from sequences and Pangolin nomenclature for lineage designations of SARS-CoV-2. Finally, we conducted a 1-sided *t* test to determine whether regional weekly speed or transmission rate per 100,000 population was greater than an outbreak threshold of 10. We ran the test iteratively with 6 months of data across the sample period.

**Results:**

Speed for the region had remained below the outbreak threshold for over a year by the time of the WHO declaration. Acceleration and jerk were also low and stable. While the 1-day persistence coefficients remained statistically significant and positive (1.168), the 7-day persistence coefficient was negative (–0.185), suggesting limited cluster effects in which cases on a given day predict cases 7 days forward. Furthermore, the shift parameters for either of the 2 most recent weeks around May 5, 2023, did not indicate any overall change in the persistence measure around the time of the WHO declaration. From December of 2021 onward, Omicron was the predominant variant of concern in sequenced viral samples. The rolling *t* test of speed equal to 10 was statistically insignificant across the entire pandemic.

**Conclusions:**

While COVID-19 continued to circulate in South Asia, the rate of transmission had remained below the outbreak threshold for well over a year ahead of the WHO declaration. COVID-19 is endemic in the region and no longer reaches the threshold of the pandemic definition. Both standard and enhanced surveillance metrics confirm that the pandemic had ended by the time of the WHO declaration. Prevention policies should be a focus ahead of future pandemics. On that point, policy should emphasize an epidemiological task force with widespread testing and a contact-tracing system.

## Introduction

COVID-19, the disease caused by the virus SARS-CoV-2, was first detected in Wuhan, China, in the fall of 2019 [1-5]. The first cases of COVID-19 in South Asia were reported in India on January 30, 2020. Our research team conducted an analysis of the pandemic in South Asia 1 year into the pandemic [6]. This study provides 2 additional years of updated surveillance and analysis for the region.

We adopt the World Bank’s definition of South Asia, which is based on economic development and geographical proximity, encompassing Afghanistan, Bangladesh, Bhutan, India, the Maldives, Nepal, Pakistan, and Sri Lanka [7].

The World Health Organization (WHO) and Director-General Tedros Adhanom Ghebreyesus declared the end of COVID-19 as a public health emergency of international concern on May 5, 2023 [8-10], based on the recommendation of the COVID-19 Emergency Committee [10]. We compare how the pandemic was progressing before and after the declaration.

Epidemiological terms, such as *pandemic*, *epidemic*, *outbreak*, and *endemic*, are used to describe the occurrence and spread of diseases [11,12]. The distinctions between these terms lie in their scope, geographic extent, and severity. An *epidemic* refers to a sudden increase in the number of disease cases in a specific population or region. If the epidemic spreads across several countries or continents, it becomes a *pandemic*. An *outbreak*, on the other hand, describes a sudden increase in a concentrated setting, usually involving a more limited geographic area than an epidemic. *Endemic* refers to the constant presence of a disease in a particular geographic region or population, with no sudden increases in case volume [13,14].

Public health surveillance is the “ongoing, systematic collection, analysis, and interpretation of health-related data essential to planning and evaluation of public health practice” [15]. Surveillance not only explains the burden of death and disease, but it generates research questions and guides researchers on topics that require further investigation [16-30]. Surveillance allows us to compare the burden of disease between geographic regions and to understand which regions are most impacted. The impact can be measured through rates of how many people contract a disease, how many die, and the affiliated costs.

Traditional surveillance carries several limitations that this study will address. Traditional surveillance provides a snapshot of what has already happened [16-30], meaning surveillance is static and focuses on the past. In the middle of a burgeoning pandemic, policy makers and public health practitioners also need to understand what is about to happen. Is an outbreak increasing? Will growth switch from linear to exponential? Are more people dying from that particular condition in one place than another? To inform health policy and practice, knowledge of what is about to happen is often more valuable than knowledge of what did happen. To that end, we have developed enhanced surveillance metrics that reflect the dynamics of a pandemic and inform imminent growth—most importantly, where along the epidemiological outbreak curve a particular region is situated. We also include dynamic metrics about the speed of the pandemic at the national, regional, and global level. We measure how acceleration of speed this week compares to last week, as well as how novel infections last week predict new cases this week. We can think of the latter measure as the echoing forward of cases. These metrics were tested and validated in prior research [31-42].

The novel indicators go beyond transmission rates to include acceleration, jerk, and 1- and 7-day persistence metrics. The transmission rate of new COVID-19 cases per 100,000 population is also known as the “speed” of the pandemic. The difference in speed from one time unit to another is called acceleration, which can identify whether the rate of new cases is increasing (positive acceleration), decreasing (negative acceleration), or stable (zero acceleration). With terminology from physics, “jerk” is the change in acceleration from one time unit to another, and a high jerk can indicate an explosive outbreak of disease. Lastly, 1- and 7-day persistence measures estimate the statistical impact of 1- and 7-day lagged speed on current speed. These two metrics are coefficient estimates from a dynamic panel data model [43].

In earlier work, this research team used the novel indicators to analyze the impact of reopening the economy on COVID-19 transmissions [41]. The metrics also assessed the status of the pandemic by global region, and they were used in policy briefs for public health decision-makers through the pandemic [44].

For the purposes of this study, standard surveillance metrics are those that explain what has already happened in South Asia, while enhanced surveillance metrics speak to what is about to happen or where along an epidemiological curve a country sits. We use both types of metrics to analyze the possible end to the pandemic in South Asia.

This study has 3 objectives. First, we aim to measure whether there was an expansion or contraction in the pandemic in South Asia when the WHO declared the end of the COVID-19 pandemic as a public health emergency of international concern on May 5, 2023 [8-10]. At both the region and country level, we use advanced surveillance and analytical techniques to describe the status of the pandemic in a 2-week window around the WHO declaration. From a public health perspective, we need to know whether the rate of new COVID-19 cases was increasing, decreasing, or stable from week to week, and if any changes in the transmission rate indicated an acceleration or deceleration of the pandemic. Statistical insignificance is significant—it can signal the epidemiological “end” to the pandemic if the rate of new cases is zero (or very low) and stable, meaning the number of new cases is neither accelerating nor decelerating.

Second, we use dynamic and genomic surveillance methods to describe the history of the pandemic in the region and situate the time window around the WHO declaration within the broader history. We include the ratio of COVID-19 deaths to the number of transmissions as a proxy for the mortality risk from infection at the population level. We also include a historical record of genomic surveillance from sequenced viral specimens to identify the appearance and spread of variants of concern (VOCs) in the region.

Third, we aim to provide historical context for the course of the pandemic in South Asia. We address several questions: How did countries respond to the pandemic? How did the region fare in terms of disease burden? What social, economic, and political factors shaped the course of COVID-19 in the region? This context can provide important lessons for disease prevention and mitigation in future pandemics.

## Methods

### Data Source

This study conducted trend analyses with longitudinal COVID-19 data from Our World in Data (OWID) [45]. OWID compiles data on COVID-19 cases and mortality from various sources, including individual websites, statistical reports, and press releases. This study provides updates for the original study by Welch et al [6] for traditional surveillance data and dynamic panel estimates [40,41,46,47]. For the region of South Asia, the data comprised an unbalanced panel of 8 countries and territories, running from August 14, 2020, to May 12, 2023. Because a number of countries around the world switched from daily to weekly reports at various points in 2023, we used a cubic spline to interpolate daily new cases and deaths if any country had 4 consecutive periods of nonzero new cases interspersed by 6 days of zero new cases.

To identify the appearance and duration of VOCs, we also used data on sequenced SARS-CoV-2 variants from the Global Initiative on Sharing All Influenza Data (GISAID), an effective and trusted web-based resource for sharing genetic, clinical, and epidemiological COVID-19 data [48-51]. We used Nextclade nomenclature [52] to collect clade designations from sequences and Pangolin nomenclature for lineage designations of SARS-CoV-2 [53,54]. Metadata for the location of the lab submitting individual specimens were accessed on June 22, 2023. To avoid low frequency or potentially erroneous samples, the data set was further filtered to exclude months with fewer than 100 available samples, variant groups with fewer than 5 samples in a month, and variant groups representing less than 0.5% of the total samples in a month. The final data set consisted of 184,386 total samples available on GISAID [48-51].

We analyzed the potential “statistical end” to the pandemic with a 1-sided *t* test for whether the mean of speed was equal to or greater than the outbreak threshold of 10. We ran the test on a rolling 6-month window over weekly speed for the region, and we plotted the *P* values from the test over time. All statistical analyses were conducted in R (version 4.2.1; R Foundation for Statistical Computing) with the *plm* package (version 2.6-2) [46].

### Ethical Considerations

The data in this study are publicly available and contain no identifiable or private information. As defined by the 45CFR46:102 policy, the study does not qualify as human subjects research. Sources have been presented in the Data Availability statement.

## Results

Table 1 presents the dynamic panel estimates for the most recent time window. The Wald test for the regression was significant (*P*<.001), and the Sargan test failed to reject the validity of the overidentification restrictions (*P*>.99). While the 1-day lag coefficient was statistically significant and positive (1.168), suggesting a cluster effect in which cases on a given day impact cases the next day, the broader persistence measure of the 7-day coefficient was negative (–0.185). Furthermore, the shift parameters for the most recent week were negative, meaning the clustering effect had become smaller in the week after the WHO declaration (but the shift parameter was positive and similar in magnitude for the prior week).

**Table 1:** Arellano-Bond dynamic panel data estimates of COVID-19 infections for South Asian countries from April 28 to May 12, 2023a.

Variable	Value	*P* value
1-day persistence coefficient	1.168	<.001
7-day persistence coefficient	–0.185	<.001
Shift parameter for week of April 28	0.052	.03
Shift parameter for week of May 5	–0.044	.13
Weekend	0.051	.33

^a^Wald test: *χ*^2^_6_>2.22e9 (*P*≤2.22e-16); Sargan: *χ*^2^_540_=8 (*P*>.99).

The dynamic panel estimates are motivated by limitations in the reproductive number, R_0_, which is the average number of people 1 contagious person will infect [55]. The central limitation is that R_0_ is influenced by many factors, such as individual behavior, vaccination rates, population density, and the transmissibility of a pathogen. Because the SARS-CoV-2 virus mutated many times, so has its R_0_, but continually updated estimates for R_0_ are difficult to obtain, as R_0_ depends not only on the transmissibility of SARS-CoV-2 but various other factors. Other factors, such as public health campaigns, have also evolved over time. The dynamic panel estimates are derived from a rolling 120-day window, so they adjust rapidly to new circumstances. The Arellano-Bond model is also robust to time-invariant, unobservable factors (ie, any stable differences between countries over the sample period), corrects for autocorrelation, and allows for statistical tests of model parameters [41].

The validity of the dynamic panel model can be partly assessed through the Wald and Sargan statistical tests. The former test checks whether the independent variables collectively have explanatory power for movements in the dependent variable. The Wald test was highly statistically significant (*P*<.001), implying a rejection of the null hypothesis that the independent variables do not explain the dependent variable. The Sargan test instead assesses the validity of the overidentifying restrictions assumed in the estimation of the model. Here, a rejection of the null would instead be evidence against the validity, but the test failed to reject with a *P* value approaching 1.

Static surveillance metrics for the weeks of April 28 and May 5, 2023, are provided in Tables 2 and 3. Aside from the Maldives, every country had a small number of new COVID-19 cases relative to the population. The highest transmission rate was observed in Afghanistan, where speed was 0.48 in the week of April 28 and 0.57 the following week. This speed was below the threshold considered a low transmission rate by the US Centers for Disease Control and Prevention (CDC) [31-42,56]. Specifically, a “low” transmission is considered no more than 10 cases per 100,000 people per week. “Moderate” transmission is 10 to 50 cases per 100,000 people per week. “Substantial” transmission is 50 to 100 [56,57].

**Table 2:** Static COVID-19 surveillance metrics for South Asian countries in the week of April 28, 2023.

Country	New COVID-19 cases, n	Cumulative COVID-19 cases, n	7-day moving average of new cases	Weekly transmission rate per 100,000 population	New deaths, n	Cumulative deaths, n	7-day moving average of deaths	Death rate per 100,000 individuals	Conditional death rate
Afghanistan	198	216,396	252.57	0.48	1	7894	0.29	0	0.04
Bangladesh	9	2,038,300	13.29	0.01	0	29,446	0	0	0.01
Bhutan	0	62,666	0.14	0	0	21	0	0	0
India	3611	44,964,289	4563.57	0.25	36	531,642	24.86	0	0.01
Maldives	15	186,435	21.57	19.76	0	313	0	0.20	0
Nepal	17	1,003,081	23.14	0.06	0	12,031	0.29	0	0.01
Pakistan	0	1,580,631	0	0	0	30,656	0	0	0.02
Sri Lanka	6	672,194	5.86	0.03	0	16,844	0.43	0	0.03

**Table 3:** Static COVID-19 surveillance metrics for South Asian countries in the week of May 5, 2023.

Country	New COVID-19 cases, n	Cumulative COVID-19 cases, n	7-day moving average of new cases	Weekly transmission rate per 100,000 population	New deaths, n	Cumulative deaths, n	7-day moving average of deaths	Death rate per 100,000 individuals	Conditional death rate
Afghanistan	235	218,454	294	0.57	3	7907	1.86	0.01	0.04
Bangladesh	23	2,038,453	21.86	0.01	0	29,446	0	0	0.01
Bhutan	0	62,668	0	0	0	21	0	0	0
India	1580	44,978,179	1984.29	0.11	17	531,753	15.86	0	0.01
Maldives	9	186,526	10.57	12.36	0	314	0.14	0.15	0
Nepal	21	1,003,205	17.71	0.07	0	12,031	0	0	0.01
Pakistan	0	1,580,631	0	0	0	30,656	0	0	0.02
Sri Lanka	24	672,265	10.14	0.11	0	16,851	1	0	0.03

Speed in the Maldives was 19 in the week of April 28 and 12 in the subsequent week. This rate of novel transmissions qualifies as a moderate outbreak, but we note that transmission rates often vacillate between high and low values in island nations. Based on the definition of a pandemic or an outbreak in several countries, the data indicate a shift from pandemic to endemic COVID-19 in South Asia, while it was epidemic in the Maldives.

A comparison of Tables 2 and 3 demonstrates little to no change before and after the WHO declared an end to the pandemic. Without question, India had the most cases of COVID-19 transmissions and deaths, but this rank is a function of population size. Thus, a better measure is the number of COVID-19 cases and deaths per 100,000 population. Moreover, death is often a better proxy for the state of an outbreak than transmissions because deaths are less likely to be undercounted [58]. Undercounting may be due to poor public health infrastructure, home antigen testing, or a dearth of polymerase chain reaction testing or other resources. While India reported 0.01 deaths per confirmed infection, several countries had higher rates. Afghanistan had the highest rate at 0.04, followed by Sri Lanka at 0.03 and Pakistan at 0.02. The relative risk of death per infection was modest in India compared to the region.

Tables 4 and 5 contain enhanced dynamic surveillance metrics for the 2 weeks before and after May 5. Again, speed was low for every country except the Maldives. Acceleration and jerk were both either small or negative for every country and territory, including the Maldives. The 7-day persistence effect on speed was also negative, suggesting a further reduction in the risk of outbreaks. These metrics suggest the pandemic may have indeed ended for the region. Because only a single territory was in a moderate outbreak, epidemiologically, COVID-19 would be considered an epidemic in the Maldives and not reach the threshold of a pandemic. We note that the figures in Tables 4 and 5 are not calculated as day-over-day averages across the week, as they are in Tables 2 and 3. Thus, the magnitudes of speed may not exactly match those in Tables 2 and 3.

**Figure 1:**
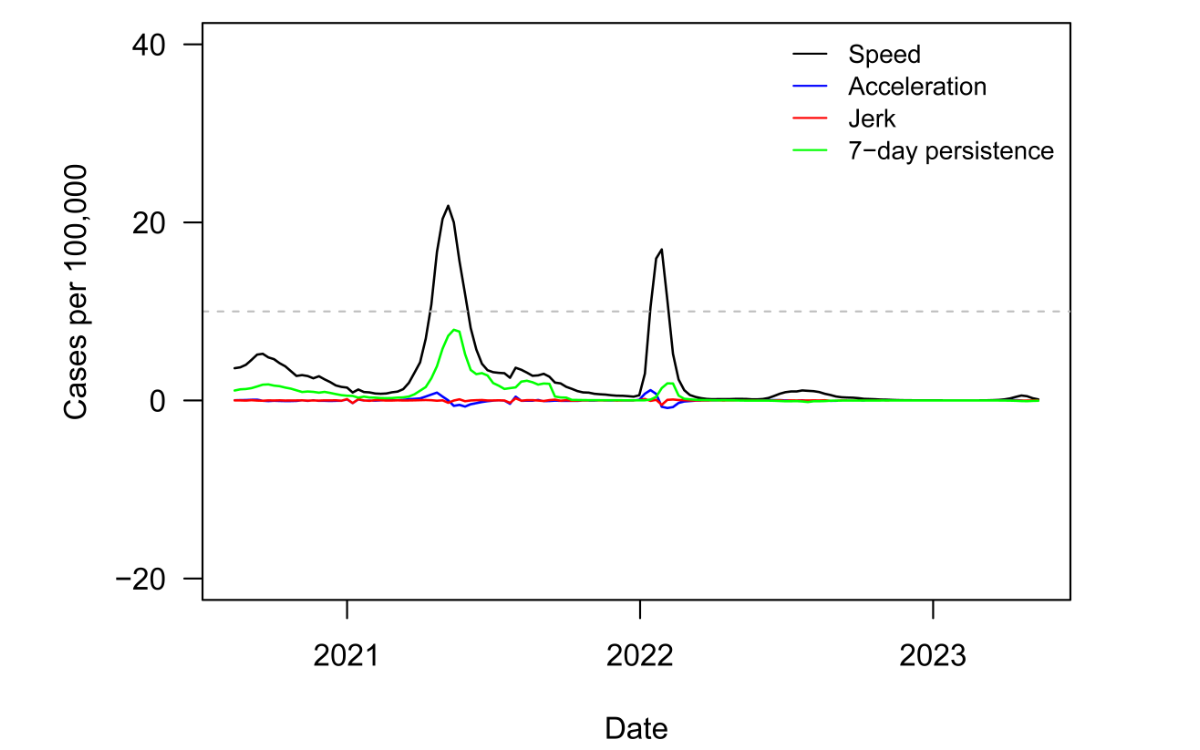
Novel surveillance metrics (speed, acceleration, jerk, 7-day persistence) for COVID-19 infections in South Asian countries from August 2020 to May 2023. The dashed gray line denotes the informal US Centers for Disease Control and Prevention outbreak threshold of speed equal to 10.

Figure 1 plots regional speed, acceleration, jerk, and 7-day persistence metrics from August 14, 2020, to May 12, 2023. The dashed gray line denotes the informal CDC outbreak threshold of speed equal to 10. In terms of disease burden, South Asia was less affected by the pandemic than most other global regions. South Asia experienced 2 relatively small outbreaks. The first began in April of 2021, lasting less than 2 months and bringing a peak speed of 22 new COVID-19 cases per 100,000 population. The second, slightly smaller outbreak began in January of 2022, lasted only a month, and brought a peak speed of 17. The region has had low and stable speed ever since.

**Table 4:** Novel COVID-19 surveillance metrics for South Asian countries in the week of April 28, 2023.

Country	Speed	Acceleration	Jerk	7-day persistence effect on speed
Afghanistan	0.61	–0.04	–0.14	–0.03
Bangladesh	0.01	0	0	0
Bhutan	0.05	0	0	–0.02
India	0.32	–0.04	0.01	–0.06
Maldives	28.70	–2.70	–0.27	–3.87
Nepal	0.08	–0.02	0.01	–0.02
Pakistan	0	0	0	0
Sri Lanka	0.03	0	0	0

**Table 5:** Novel COVID-19 surveillance metrics for South Asian countries in the week of May 5, 2023.

Country	Speed	Acceleration	Jerk	7-day persistence effect on speed
Afghanistan	0.71	0.01	0.07	–0.08
Bangladesh	0.01	0	0	0
Bhutan	0.04	0	0	–0.01
India	0.14	–0.02	0	–0.04
Maldives	14.12	–1.06	0.37	–3.83
Nepal	0.06	0	0.01	–0.01
Pakistan	0	0	0	0
Sri Lanka	0.05	0.01	0.01	0

**Figure 2:**
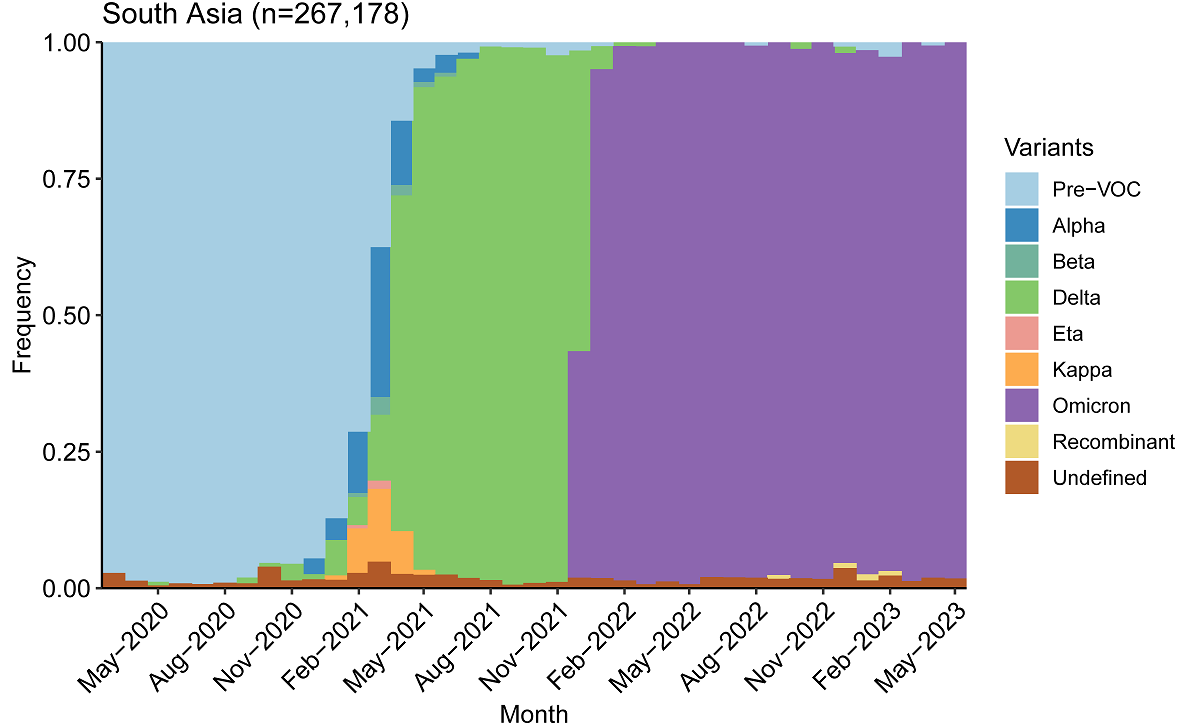
Variant groups as a proportion of all sequenced SARS-CoV-2 specimens from March 2020 to May 2023 in South Asia. VOC: variant of concern.

Figure 2 plots variant groups as a proportion of all viral specimens collected and sequenced in the region (and made available through GISAID) each month. Matching the timeline to Figure 1, the first regional outbreak was driven by the Delta variant, while the second outbreak was driven by Omicron. South Asia, like much of the rest of the world, saw a surge in cases amid the heightened transmissibility of Omicron [44]. However, the surge was much smaller in South Asia than in most other parts of the world.

Another potential indication of the end to the pandemic is the continued dominance of the Omicron variant. While the region saw a mixture of variants prior to the arrival of Omicron in December of 2021, viral sequences have almost exclusively returned as Omicron and its subvariants ever since.

**Figure 3:**
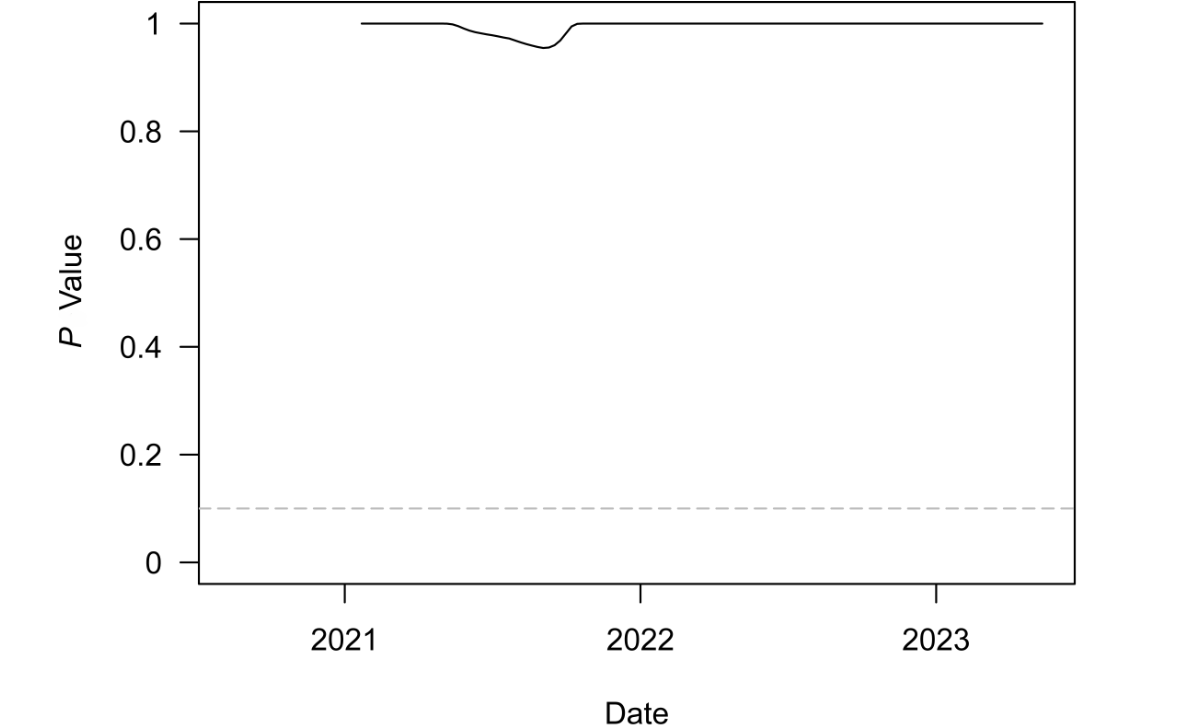
*P* values from t tests of weekly COVID-19 transmissions per 100,000 population equal to 10 over a rolling 6-month window in South Asia. The dashed gray line denotes the least restrictive conventional significance level threshold of α=.10.

Figure 3 plots *P* values from a series of 1-sided *t* tests to determine whether speed for the region was equal to or greater than the outbreak threshold of 10. These tests were conducted on a rolling 6-month window of weekly regional speed. The dashed gray line denotes the least restrictive conventional significance level threshold of α=.10. The test never rejected the null in favor of the alternative. In fact, the test statistic was totally insignificant outside of a brief, tiny dip in the *P* value over the period covering the Delta-driven outbreak. The continued lack of statistical significance ever since is consistent with the end to the pandemic in the region, as the test clearly failed to reject the null hypothesis that the weekly speed or transmission rate was lower than the outbreak threshold.

**Figure 4:**
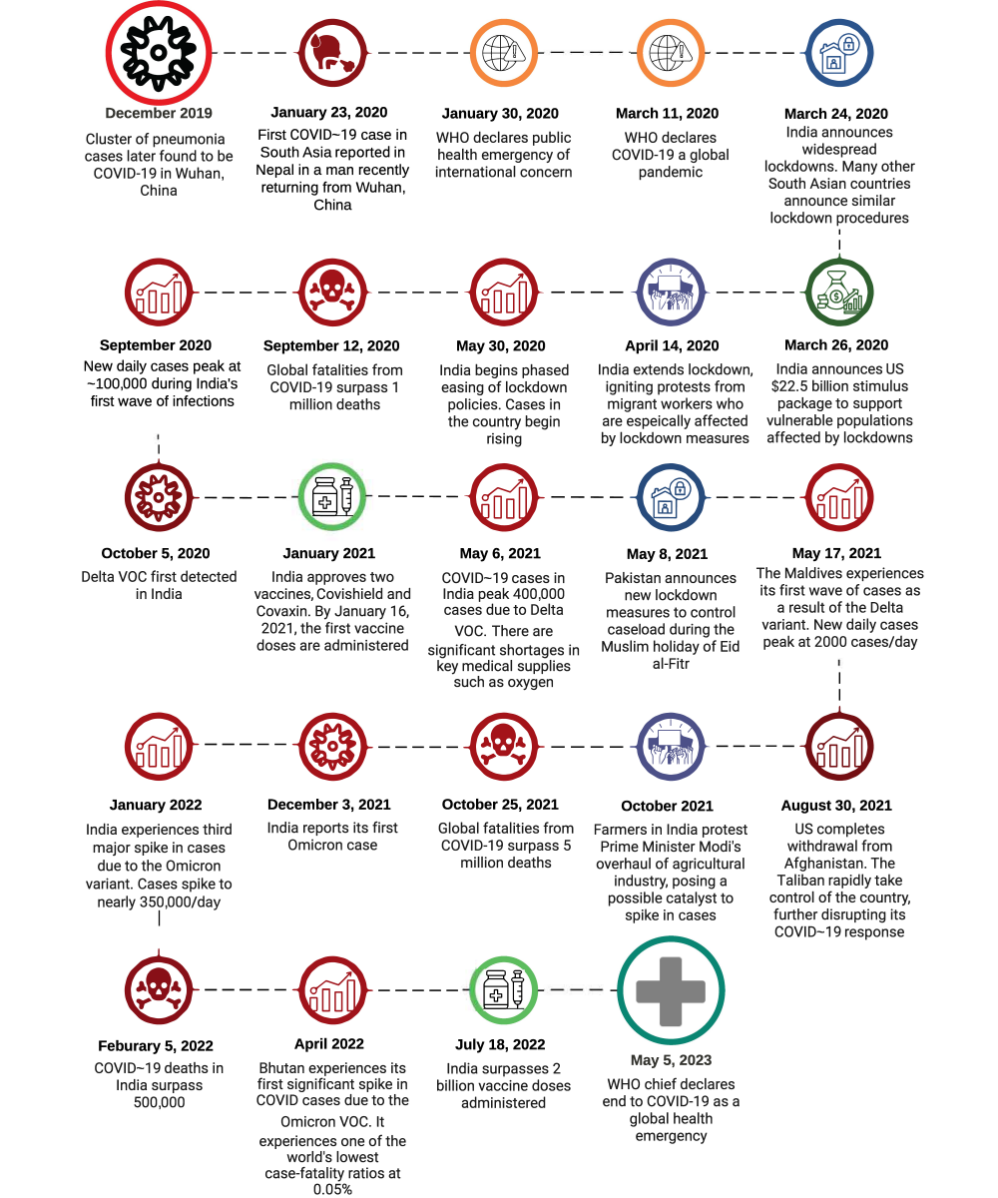
Timeline of the COVID-19 pandemic in South Asia. VOC: variant of concern; WHO: World Health Organization.

Figure 4 provides a timeline of the onset of COVID-19 in South Asia, as well as vaccination programs and major events that shaped the course of the pandemic in the region, such as economic measures and political conflict.

## Discussion

### Principal Findings

While COVID-19 continues to circulate in South Asia, the rate of transmission had consistently remained below the outbreak threshold for well over a year prior to the WHO declaration. COVID-19 is endemic in the region, no longer meeting the criteria for pandemic classification. There has been neither significant acceleration nor deceleration in the region, and transmission rates remained below the threshold of an outbreak. Finally, the statistical echo-forward effect of COVID-19 cases on future cases had dissipated well before the WHO declared the end of the pandemic public health emergency. Both standard and enhanced surveillance metrics confirm that the pandemic had concluded by the time of the WHO declaration.

South Asia experienced independent rather than overlapping waves of COVID-19 [59]. The initial wave of COVID-19 in South Asia, while milder in caseload compared to Europe and the Americas, was influenced by a large population of migrant workers who moved across regions, increasing transmission risk [60]. Lockdowns led to reverse migration, facilitating virus spread [60,61]. India, Bangladesh, and Pakistan are home to 139 million migrant workers who traveled out of their home states for work [62]. As countries in the region instituted lockdowns, unemployed migrants [61-64] “reverse migrated” back to their residence of origin, creating a natural conduit for COVID-19 to spread in South Asia [60,65].

The second wave in early 2021 was severe, driven by the Delta variant. India reported over 400,000 daily cases, straining health care systems and causing oxygen shortages [66-68]. Due to high demand and a lack of health care infrastructure, many COVID-19 patients did not receive adequate treatment, resulting in higher mortality. Crematoriums and burial grounds were quickly overwhelmed [66,69-71]. Bangladesh, Pakistan, and Sri Lanka also experienced a rise in cases and deaths from the Delta-variant wave [72-75]. Afghanistan grappled with significant sociopolitical instability following the withdrawal of US troops from the country and the government takeover by the Taliban [76,77]. Still, South Asia fared relatively better than other regions, with lower transmission rates.

Economically, South Asia’s gross domestic product (GDP) contracted by 5.2% in 2020 due to lockdowns, with India’s GDP declining by 24.4% in Q2 2020 [78,79]. Governments implemented stimulus packages, including cash support, corporate incentives, and health care investments [75,76]. As vaccines became available, economies began recovering, with the region’s GDP growing by 8% in 2021 [78-80].

### Policies Implemented to Control and Mitigate the Transmission of COVID-19

COVID-19 containment strategies in South Asia included masking, border closures, contact tracing, social distancing, quarantines, and lockdowns. India’s phased reopening faced challenges [79]. Pakistan attempted to ease restrictions in April 2020 and had to partially reverse course due to a second wave of infections [79]. Afghanistan struggled due to health care limitations and political instability [76].

South Asian countries aimed for herd immunity through vaccination. India initiated a massive vaccination campaign in January 2021 [81]. Supply chain issues affected progress, contributing to the Delta variant’s impact [82]. Vaccine hesitancy prompted public health campaigns in some countries [83-85].

Even though South Asia has shifted from pandemic to endemic, there is a possibility that a novel VOC could potentially be more transmissible, resistant to vaccines, or cause more severe illness. This underlines the importance of continued vigilance, vaccination efforts, and global cooperation to control the spread of the virus. [39].

### Limitations

COVID-19 data had become less frequently reported around the world by the time the WHO declared an end to the pandemic public health emergency [86]. Additionally, more people began to use at-home tests as the pandemic evolved, leading to an undercount of cases [87]. Because the enhanced surveillance metrics of speed, acceleration, jerk, and 7-day persistence are based on rates, not total counts, statistical bias caused by countries dropping in or out of the sample is mitigated, but to the extent that a nonincluded country is unrepresentative of the region in disease burden, the omission of a country or territory can still influence historical data comparisons. Viral specimen tests for VOCs in GISAID are also dependent on testing and sequencing capacity, which varies by country across the region.

### Conclusion

Although South Asia experienced only 2 brief outbreaks of COVID-19 during the pandemic, its disease burden was still somewhat high due to its population size, with well over 500,000 deaths. One of the most important lessons from the COVID-19 pandemic is preparedness for future pandemics. At the country level, an epidemiological task force with rapid, widespread testing capacity and a contact-tracing system should be prioritized [88]. Lockdown policies are effective but may have heightened economic costs in less economically developed countries [89]. Indicators of preparedness from a regional perspective might help identify countries in need of support, as measures of governance are positively associated with, for example, vaccination rates [90,91]. Thus, cooperation at the regional level will be a continued necessity for effective disease mitigation in future pandemics [92,93].

## Abbreviations

CDC: US Centers for Disease Control and Prevention
GDP: gross domestic product
GISAID: Global Initiative on Sharing All Influenza Data
OWID: Our World in Data
VOC: variant of concern
WHO: World Health Organization

## Data Availability

This study relied on publicly available data from 2 sources. Data on COVID-19 transmissions and death rates came from Our World in Data [45]. Data on sequenced SARS-CoV-2 specimens came from the Global Initiative on Sharing All Influenza Data (GISAID) [48].

## References

[1] Muralidar S, Ambi SV, Sekaran S, Krishnan UM (2020). The emergence of COVID-19 as a global pandemic: Understanding the epidemiology, immune response and potential therapeutic targets of SARS-CoV-2. Biochimie.

[2] Sharma A, Ahmad Farouk I, Lal SK (2021). COVID-19: a review on the novel coronavirus disease evolution, transmission, detection, control and prevention. Viruses.

[3] Chilamakuri R, Agarwal S (2021). COVID-19: characteristics and therapeutics. Cells.

[4] Hu B, Guo H, Zhou P, Shi Z (2021). Characteristics of SARS-CoV-2 and COVID-19. Nat Rev Microbiol.

[5] Seyed Hosseini E, Riahi Kashani N, Nikzad H, Azadbakht J, Hassani Bafrani H, Haddad Kashani H (2020). The novel coronavirus disease-2019 (COVID-19): mechanism of action, detection and recent therapeutic strategies. Virology.

[6] Welch SB, Kulasekere DA, Prasad PVV, Moss CB, Murphy RL, Achenbach CJ, Ison MG, Resnick D, Singh L, White J, Issa TZ, Culler K, Boctor MJ, Mason M, Oehmke JF, Faber JMM, Post LA (2021). The interplay between policy and COVID-19 outbreaks in South Asia: longitudinal trend analysis of surveillance data. JMIR Public Health Surveill.

[7] The world by region. World Bank.

[8] Smith-Schoenwalder C (2023). When does the COVID-19 pandemic end?. U.S. News and World Report.

[9] Burki T (2023). WHO ends the COVID-19 public health emergency. Lancet Respir Med.

[10] (2023). WHO chief declares end to COVID-19 as a global health emergency. United Nations.

[11] (2012). Lesson 1: introduction to epidemiology. US Centers for Disease Control and Prevention.

[12] Epidemic, endemic, pandemic: what are the differences?. Columbia University.

[13] Lancet Infectious Diseases (2022). Transitioning to endemicity with COVID-19 research. Lancet Infect Dis.

[14] Britto D Pandemic to endemic: the race against time. Tony Blair Institute for Global Change.

[15] Hong R, Walker Rebecca, Hovan Gregory, Henry Lisa, Pescatore Rick (2020). The power of public health surveillance. Dela J Public Health.

[16] Teutsch SM, Churchill RE (2000). Principles and Practice of Public Health Surveillance.

[17] Teutsch SM, Lee LM, Teutsch SM, Thacker SB, St. Louis ME (2010). Considerations in planning a surveillance system. Principles & Practice of Public Health Surveillance, 3rd Ed.

[18] Teutsch SM, Thacker SB (1995). Planning a public health surveillance system. Epidemiol Bull.

[19] Thacker S, Qualters Judith R, Lee Lisa M (2012). Public health surveillance in the United States: evolution and challenges. MMWR Suppl.

[20] Centers for Disease Control (CDC) (1988). Guidelines for evaluating surveillance systems. MMWR Suppl.

[21] Lee LM, Thacker SB (2011). Public health surveillance and knowing about health in the context of growing sources of health data. Am J Prev Med.

[22] Davis Am, Dunet Do, Keaton R, Snider De (1996). CDC guidelines: improving the quality. US Centers for Disease Control and Prevention.

[23] Thacker S, Stroup D F (1994). Future directions for comprehensive public health surveillance and health information systems in the United States. Am J Epidemiol.

[24] Nsubuga P, White M, Thacker S, Anderson M, Blount S, Broome C, Chiller T, Espitia V, Imtiaz R, Sosin D, Stroup D, Tauxe R, Vijayaraghavan M, Trostle M, Jamison D, Breman J, Measham A, Alleyne G, Claeson M, Evans D, Jha P, Mills A, Musgrove P (2006). Public health surveillance: a tool for targeting and monitoring interventions. Disease Control Priorities in Developing Countries. 2nd Edition.

[25] Lee L, Teutsch S, Thacker S, St. Louis M (2010). Principles and Practice of Public Health Surveillance. 3rd Edition.

[26] Thacker SB, Stroup DF, Rothenberg RB (1995). Public health surveillance for chronic conditions: a scientific basis for decisions. Stat Med.

[27] Perry HN, McDonnell SM, Alemu W, Nsubuga P, Chungong S, Otten MW, Lusamba-dikassa PS, Thacker SB (2007). Planning an integrated disease surveillance and response system: a matrix of skills and activities. BMC Med.

[28] Koo D, Thacker SB (2010). In snow's footsteps: Commentary on shoe-leather and applied epidemiology. Am J Epidemiol.

[29] Romaguera R, German RR, Klaucke DN, Teutsch SM, Churchill RE (2000). Evaluating public health surveillance. Principles and Practice of Public Health Surveillance.

[30] Pappaioanou M, Malison M, Wilkins K, Otto B, Goodman RA, Churchill R, White M, Thacker SB (2003). Strengthening capacity in developing countries for evidence-based public health: the data for decision-making project. Soc Sci Med.

[31] Post L, Boctor MJ, Issa TZ, Moss CB, Murphy RL, Achenbach CJ, Ison MG, Resnick D, Singh L, White J, Welch SB, Oehmke JF (2021). SARS-CoV-2 surveillance system in Canada: longitudinal trend analysis. JMIR Public Health Surveill.

[32] Post L, Culler K, Moss CB, Murphy RL, Achenbach CJ, Ison MG, Resnick D, Singh LN, White J, Boctor MJ, Welch SB, Oehmke JF (2021). Surveillance of the second wave of COVID-19 in Europe: longitudinal trend analyses. JMIR Public Health Surveill.

[33] Post L, Marogi E, Moss CB, Murphy RL, Ison MG, Achenbach CJ, Resnick D, Singh L, White J, Boctor MJ, Welch SB, Oehmke JF (2021). SARS-CoV-2 surveillance in the Middle East and North Africa: longitudinal trend analysis. J Med Internet Res.

[34] Post L, Ohiomoba RO, Maras A, Watts SJ, Moss CB, Murphy RL, Ison MG, Achenbach CJ, Resnick D, Singh LN, White J, Chaudhury AS, Boctor MJ, Welch SB, Oehmke JF (2021). Latin America and the Caribbean SARS-CoV-2 surveillance: longitudinal trend analysis. JMIR Public Health Surveill.

[35] Post LA, Argaw ST, Jones C, Moss CB, Resnick D, Singh LN, Murphy RL, Achenbach CJ, White J, Issa TZ, Boctor MJ, Oehmke JF (2020). A SARS-CoV-2 surveillance system in sub-Saharan Africa: modeling study for persistence and transmission to inform policy. J Med Internet Res.

[36] Post LA, Benishay ET, Moss CB, Murphy RL, Achenbach CJ, Ison MG, Resnick D, Singh LN, White J, Chaudhury AS, Boctor MJ, Welch SB, Oehmke JF (2021). Surveillance metrics of SARS-CoV-2 transmission in Central Asia: longitudinal trend analysis. J Med Internet Res.

[37] Post LA, Issa TZ, Boctor MJ, Moss CB, Murphy RL, Ison MG, Achenbach CJ, Resnick D, Singh LN, White J, Faber JMM, Culler K, Brandt CA, Oehmke JF (2020). Dynamic public health surveillance to track and mitigate the US COVID-19 epidemic: longitudinal trend analysis study. J Med Internet Res.

[38] Post LA, Lin JS, Moss CB, Murphy RL, Ison MG, Achenbach CJ, Resnick D, Singh LN, White J, Boctor MJ, Welch SB, Oehmke JF (2021). SARS-CoV-2 wave two surveillance in East Asia and the Pacific: longitudinal trend analysis. J Med Internet Res.

[39] Post LA, Lorenzo-Redondo R (2022). Omicron: fewer adverse outcomes come with new dangers. Lancet.

[40] Oehmke JF, Moss CB, Singh LN, Oehmke TB, Post LA (2020). Dynamic panel surveillance of COVID-19 transmission in the United States to inform health policy: observational statistical study. J Med Internet Res.

[41] Oehmke JF, Oehmke TB, Singh LN, Post LA (2020). Dynamic panel estimate-based health surveillance of SARS-CoV-2 infection rates to inform public health policy: model development and validation. J Med Internet Res.

[42] Oehmke TB, Post LA, Moss CB, Issa TZ, Boctor MJ, Welch SB, Oehmke JF (2021). Dynamic panel data modeling and surveillance of COVID-19 in metropolitan areas in the United States: longitudinal trend analysis. J Med Internet Res.

[43] Arellano M, Bond S (1991). Some tests of specification for panel data: Monte Carlo evidence and an application to employment equations. Rev Econ Stud.

[44] Lundberg AL, Lorenzo-Redondo R, Ozer EA, Hawkins CA, Hultquist JF, Welch SB, Prasad PV, Oehmke JF, Achenbach CJ, Murphy RL, White JI, Havey RJ, Post LA (2022). Has Omicron changed the evolution of the pandemic?. JMIR Public Health Surveill.

[45] Mathieu E, Ritchie H, Rodés-Guirao L, Appel C, Giattino C, Hasell J, Macdonald B, Dattani S, Beltekian D, Ortiz-Ospina E, Roser M Coronavirus pandemic (COVID-19). Our World in Data.

[46] Croissant Y, Millo G (2008). Panel data econometrics in R: the plm package. J Stat Soft.

[47] Hansen LP (1982). Large sample properties of generalized method of moments estimators. Econometrica.

[48] GISAID Initiative.

[49] Khare S, Gurry Céline, Freitas Lucas, Schultz Mark B, Bach Gunter, Diallo Amadou, Akite Nancy, Ho Joses, Lee Raphael Tc, Yeo Winston, Curation Team Gisaid Core, Maurer-Stroh Sebastian (2021). GISAID's role in pandemic response. China CDC Wkly.

[50] Shu Y, McCauley John (2017). GISAID: Global initiative on sharing all influenza data - from vision to reality. Euro Surveill.

[51] Nasereddin A, Golan Berman H, Wolf DG, Oiknine-Djian E, Adar S (2022). Identification of SARS-CoV-2 variants of concern using amplicon next-generation sequencing. Microbiol Spectr.

[52] Huddleston J, Hadfield J, Sibley T, Lee J, Fay K, Ilcisin M, Harkins E, Bedford T, Neher R, Hodcroft E (2021). Augur: a bioinformatics toolkit for phylogenetic analyses of human pathogens. J Open Source Softw.

[53] Rambaut A, Holmes EC, O'Toole Áine, Hill V, McCrone JT, Ruis C, du Plessis L, Pybus OG (2020). A dynamic nomenclature proposal for SARS-CoV-2 lineages to assist genomic epidemiology. Nat Microbiol.

[54] O'Toole Áine, Scher Emily, Underwood Anthony, Jackson Ben, Hill Verity, McCrone John T, Colquhoun Rachel, Ruis Chris, Abu-Dahab Khalil, Taylor Ben, Yeats Corin, du Plessis Louis, Maloney Daniel, Medd Nathan, Attwood Stephen W, Aanensen David M, Holmes Edward C, Pybus Oliver G, Rambaut Andrew (2021). Assignment of epidemiological lineages in an emerging pandemic using the pangolin tool. Virus Evol.

[55] Chen J (2020). Pathogenicity and transmissibility of 2019-nCoV-A quick overview and comparison with other emerging viruses. Microbes Infect.

[56] Levenson E, Firger J (2021). What the CDC’s ‘substantial’ and ‘high’ levels of Covid-19 transmission actually mean. CNN.

[57] Christie A, Brooks JT, Hicks LA, Sauber-Schatz EK, Yoder JS, Honein MA, CDC COVID-19 Response Team (2021). Guidance for implementing COVID-19 prevention strategies in the context of varying community transmission levels and vaccination coverage. MMWR Morb Mortal Wkly Rep.

[58] Stoto M (1992). Public health assessment in the 1990s. Annu Rev Public Health.

[59] Lundberg AL, Lorenzo-Redondo R, Hultquist JF, Hawkins CA, Ozer EA, Welch SB, Prasad PVV, Achenbach CJ, White JI, Oehmke JF, Murphy RL, Havey RJ, Post LA (2022). Overlapping Delta and Omicron outbreaks during the COVID-19 pandemic: dynamic panel data estimates. JMIR Public Health Surveill.

[60] Iyengar K, Jain Vijay Kumar (2021). COVID-19 and the plight of migrants in India. Postgrad Med J.

[61] Abi-Habib M, Yasir S (2020). India’s coronavirus lockdown leaves vast numbers stranded and hungry. The New York Times.

[62] Sharma K (2017). India has 139 million internal migrants. They must not be forgotten. World Economic Forum.

[63] Rao M (2020). Over 10 agonizing days, this migrant worker walked and hitchhiked 1,250 miles home. India’s lockdown left him no choice. CNN.

[64] Slater J, Masih N (2020). In India, the world’s biggest lockdown has forced migrants to walk hundreds of miles home. The Washington Post.

[65] Pal A (2020). Outrage in India as migrants sprayed with disinfectant to fight coronavirus. Reuters.

[66] Bhowmick N How India’s second wave became the worst COVID-19 surge in the world. National Geographic.

[67] Tian D, Sun Y, Zhou J, Ye Q (2021). The Global epidemic of the SARS-CoV-2 Delta variant, key spike mutations and immune escape. Front Immunol.

[68] McFall-Johnsen M India is the first country to record 400,000 coronavirus cases in a single day. Business Insider.

[69] India’s crematoriums overwhelmed as virus ‘swallows people’. Politico.

[70] Jamkhandikar S, Arora N Indian hospitals swamped by coronavirus as countries promise aid. Reuters.

[71] Pandey G Covid in Uttar Pradesh: coronavirus overwhelms India's most populous state. BBC.

[72] Delta variant of Covid-19 spreading fast in Sri Lanka. Times of India.

[73] Mallawarachi B Sri Lanka banks on vaccination to see it through delta surge. Medical Xpress.

[74] Samath F Sri Lanka placed on lockdown as Covid-19 cases spike. TTG Asia.

[75] Delta variant: Which Asian countries are seeing rising cases?. BBC.

[76] Essar MY, Hasan MM, Islam Z, Riaz MMA, Aborode AT, Ahmad S (2021). COVID-19 and multiple crises in Afghanistan: an urgent battle. Confl Health.

[77] Hadid D A crippling 3rd wave Of COVID adds to Afghanistan's woes. NPR.

[78] GDP growth (annual %) - South Asia. World Bank.

[79] Policy responses to COVID-19. International Monetary Fund.

[80] India highlights role of PM-GKAY scheme in ensuring food security during pandemic at WTO meet. Economic Times.

[81] Biswas S Coronavirus vaccine: India begins world's biggest drive. BBC.

[82] India is the world's biggest vaccine maker. Yet only 4% of Indians are vaccinated. NPR.

[83] Mehmood Q, Ullah I, Hasan MM, Kazmi SK, Ahmadi A, Lucero-Prisno DE (2022). COVID-19 vaccine hesitancy: Pakistan struggles to vaccinate its way out of the pandemic. Ther Adv Vaccines Immunother.

[84] Nemat A, Bahez Ayesha, Salih Mohibullah, Raufi Nahid, Noor Noor Ahmad Shah, Essar Mohammad Yasir, Ehsan Ehsanullah, Asady Abdullah (2021). Public willingness and hesitancy to take the COVID-19 vaccine in Afghanistan. Am J Trop Med Hyg.

[85] Swarnamali H, Francis Tormalli V, Sooriyaarachchi Piumika, Jayawardena Ranil (2023). COVID-19 vaccine hesitancy in Sri Lanka: A national level survey. Int J Health Sci (Qassim).

[86] Stein R (2023). As the pandemic ebbs, an influential COVID tracker shuts down. National Public Radio.

[87] Ritchey MD, Rosenblum HG, Del Guercio K, Humbard M, Santos S, Hall J, Chaitram J, Salerno RM (2022). COVID-19 self-test data: challenges and opportunities - United States, October 31, 2021-June 11, 2022. MMWR Morb Mortal Wkly Rep.

[88] Benati I, Coccia M (2022). Effective contact tracing system minimizes COVID-19 related infections and deaths: policy lessons to reduce the impact of future pandemic diseases. J Public Admin Govern.

[89] Askitas N, Tatsiramos K, Verheyden B (2021). Estimating worldwide effects of non-pharmaceutical interventions on COVID-19 incidence and population mobility patterns using a multiple-event study. Sci Rep.

[90] Coccia M (2022). Preparedness of countries to face COVID-19 pandemic crisis: strategic positioning and factors supporting effective strategies of prevention of pandemic threats. Environ Res.

[91] Benati I, Coccia M (2022). Global analysis of timely COVID-19 vaccinations: improving governance to reinforce response policies for pandemic crises. Int J Health Gov.

[92] Coccia M (2021). Pandemic prevention: lessons from COVID-19. Encyclopedia.

[93] Ear S (2021). Viral Sovereignty and the Political Economy of Pandemics.

